# Training the diagnostic artificial intelligence in thyroid sonography: how well is deep learning truly learning?

**DOI:** 10.1007/s13304-025-02506-5

**Published:** 2026-02-11

**Authors:** Oana Lozan, Petra B. Musholt, Ann-Kathrin Lederer, Nabila Bouzakri, Hauke Lang, Thomas J. Musholt

**Affiliations:** Section of Endocrine Surgery, Department of General, Visceral and Transplantation Surgery, University Medical Center Mainz, Langenbeckstraße 1, 55131 Mainz, Germany

**Keywords:** Thyroid ultrasound, Thyroid nodules, ACR TI-RADS, Artificial intelligence (AI), Deep learning, Endocrine surgery

## Abstract

**Introduction:**

The use of artificial intelligence (AI) for TI-RADS classification in neck sonography for the assessment of thyroid nodules has been proven beneficial. We evaluated the learning curve of such AI in a real-world setting.

**Methods:**

Between 03/2023 and 06/2024, 110 patients with 176 thyroid nodules were examined and classified according to ACR TI-RADS classification using 3D-ultrasound PIUR tUS Infinity software. After software training and update, the study was repeated examining 133 patients with 228 nodules (03/2023 until 10/2024). Every AI-based TI-RADS evaluation was compared to that of an experienced endocrine surgeon (assessor), unaware of the software results.

**Results:**

First phase: AI-supported TI-RADS classification corresponded to the assessor’s in 128/176 (73%) of examined nodules; second phase: correspondence in 210/227 (92.6%). Re-evaluating the initial 110 patients after software update, more nodules (194 vs. 176) were correctly assessed. In the first phase, AI “misinterpreted” in 36/176 (20%) cases nodules with microcalcifications or echogenic foci (leading to 1–3 points differences to accessor). After update, only 11/227 (4.8%) such nodules remained and relevant differences to the accessor were found in only 6/227 (2.6%) unusual cases (autoimmune thyroiditis, non-descended thymus, hemorrhaged cyst), compared to the initial 7%.

**Conclusions:**

After several rounds of deep learning, a significant improvement in correct ACR TI-RADS classification was demonstrated, especially the assessment of nodules within conglomerates. The AI-supported ultrasound is already a solid tool in the diagnosis of true thyroid nodules in non-inflamed tissue, but cannot yet replace an experienced clinician in complex or unusual cases; however, the fast learning curve is encouraging.

## Introduction

Thyroid nodules are the most frequent finding in endocrinology, with up to 65% of the general population affected [1]. The advent of high-resolution imaging, particularly ultrasound, has increased the detection of thyroid nodules and early-stage thyroid carcinoma [2], paralleled by a true rise in disease incidence [3]. Despite thyroid carcinoma being the most common endocrine malignancy encountered by surgeons, fewer than 5% of nodules are malignant, leading some authors to question the value of widespread thyroid screening [4]. Standardized risk stratification systems, such as the American College of Radiology Thyroid Imaging Reporting and Data System (ACR TI-RADS) [5], have become indispensable in differentiating benign from malignant nodules, thereby reducing the risk of overdiagnosis and overtreatment [6].

Recent development in medical imaging, particularly sonography, have notably improved the diagnosis of thyroid nodules and identifying those requiring surgical management. Accurate risk stratification remains fundamental to optimizing therapeutic decisions [5]. However, data quality issues continue to pose challenges in routine clinical practice. The incorporation of artificial intelligence (AI) into medical imaging offers an enhanced diagnostic efficiency, potentially reducing human error, a topic of growing interest across surgical and medical disciplines [7]. Increasing evidence suggest that AI systems can perform comparably to, or even exceed, human experts in sonographic evaluation of thyroid disease [8–12]. The application of deep learning methods in thyroid imaging has promising diagnostic performance [13], even beyond imaging [14]. Deep learning employs multiple layers of machine learning algorithms to analyze verified real-world data, with diagnostic accuracy improving proportionally to data volume and quality. Further progress is warranted to achieve more precise, noninvasive diagnostic modalities [15],

While AI-based systems have demonstrated benefits in settings with limited access to expert clinicians [16], their incremental value over experienced specialists remains to be confirmed. Despite these encouraging results, prospective clinical validation of such systems and their ability to improve remains limited, underscoring the need to rigorous testing under real-world conditions such as in clinical implementation.

This study aimed to analyze the initial learning curve of an AI-assisted ultrasound system using data of preoperative thyroid sonographies and verifying the sonographic diagnoses with histopathological results after (hemi)thyroidectomy. The core challenge of our study was to evaluate the extent to which deep learning is indeed capable of improving diagnostic accuracy in thyroid sonography, not only in a controlled (lab) environment, but in real-world clinical practice. This objective can be achieved by comparing the results of AI-assisted diagnostics with the clinical expertise of an experienced physician/surgeon. Furthermore, we aimed at identifying the challenges associated with the use of AI-supported software in sonography and to validate the AI-generated recommendations for clinical practice, with the overall mindset to improve the quality of patient care [6, 8–12, 15–17].

## Methods

All patients with thyroid findings who presented in the outpatient clinic of the Section of Endocrine Surgery in the Department of General, Visceral and Transplantation Surgery at the University Medical Centre Mainz, Germany, between March 2023 and October 2024, for the evaluation of thyroid surgery were included in this retrospective study, provided that routine diagnostic ultrasound data with PIUR-tUS Infinity software was collected. No specific exclusion criteria were applied (Table 1).

**Table 1:** Population examined with the AI-supported ultrasound and included in the study

Feature	No. of patients
Sex	91 females / 42 males
Age distribution	11–84 years (median, 50.5)
No surgical therapy recommended	76 (54 females / 22 males)
Patients undergoing surgery	57 (37 females / 20 males)
Benign nodules (histology result)	35 (21 females / 14 males)
Papillary thyroid carcinoma (PTC)	19 (14 females / 5 males)
Medullary thyroid carcinoma (MTC)	2 females
Poorly differentiated (PDTC) / anaplastic thyroid carcinoma	1 male

**Fig. 1:**
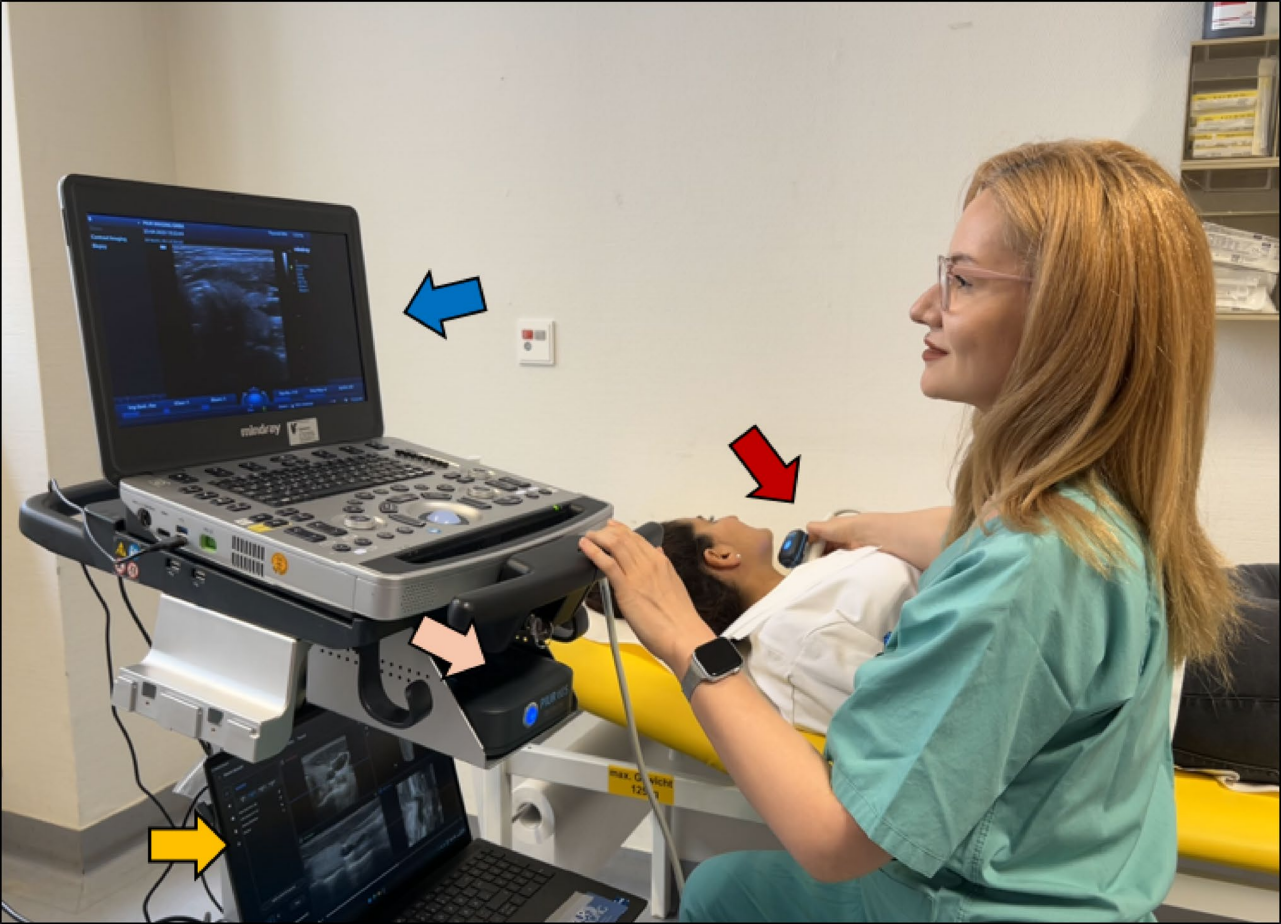
Use of AI-software during a preoperative thyroid and neck ultrasound

**Fig. 2:**
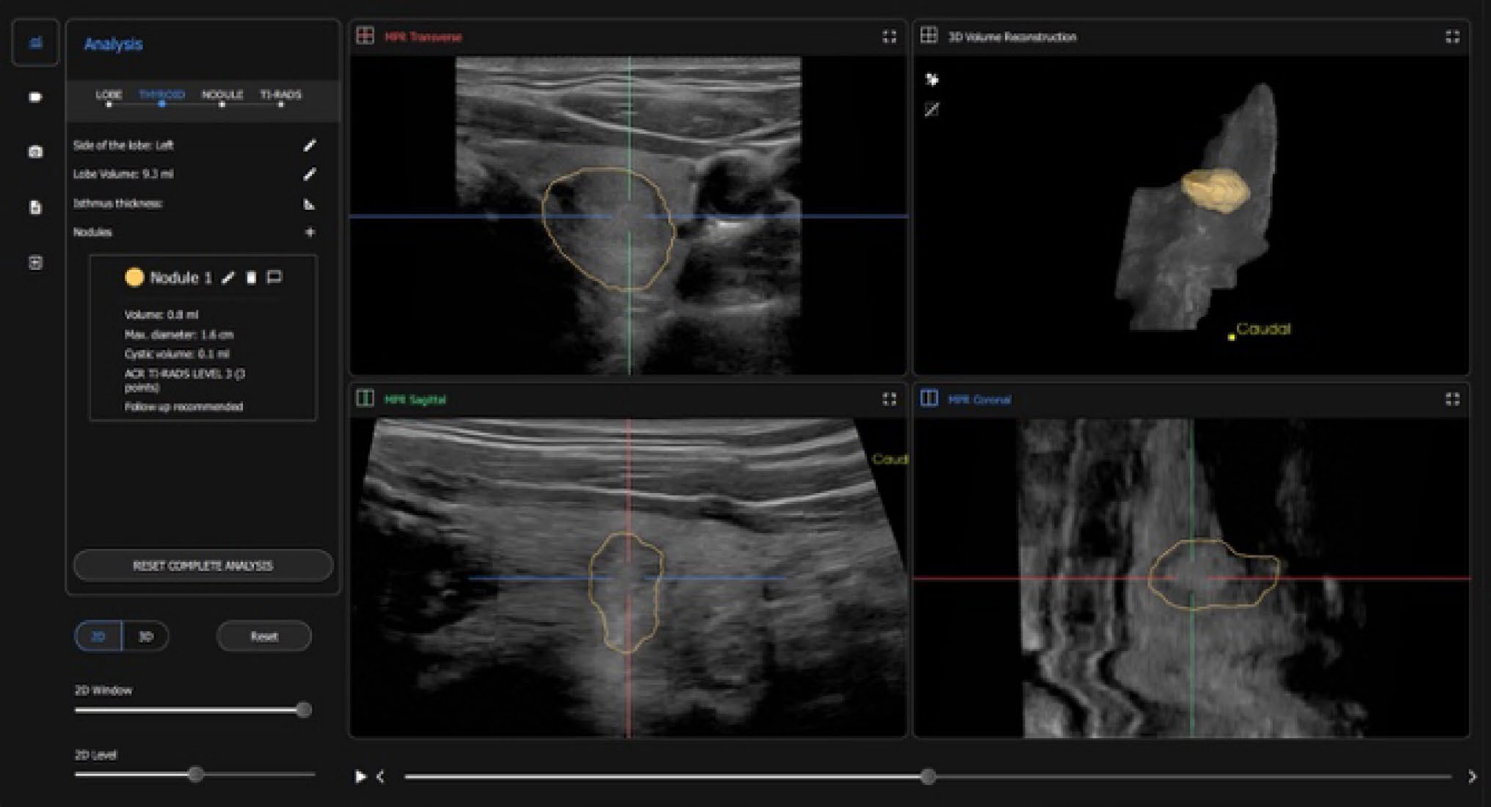
Example of an AI-assisted thyroid ultrasound examination result: 3D model of the thyroid lobe with lobe volume, detected nodules, nodule measurements, nodule volume and ACR TI-RADS assessment, as well as a recommendation for further courses of action

In the first study phase between March 2023 and June 2024, 110 patients with 176 thyroid nodules were preoperatively examined by a surgeon-in-training (resident) using the 3D-sonography and AI-based software. As usual, the ultrasound probe was placed on the neck over the thyroid gland area to obtain images. Each lobe was scanned separately and inspected for nodules. The ultrasound machine is connected to a notebook which runs the AI-assisted software, presenting the images seen on the ultrasound screen in real-time (Fig. 1). The AI-program was run and completed by a surgeon-in-training (Fig. 2). Thereafter, a highly experienced consultant endocrine surgeon (assessor) independently and without knowledge of the resident’s results, examined the patient again, assessed the nodules based on the ACR TI-RADS risk stratification system (Table 2) by his own judgement. At the end of each patient examination, the two assessments were compared and discussed for teaching purposes.

**Table 2:**
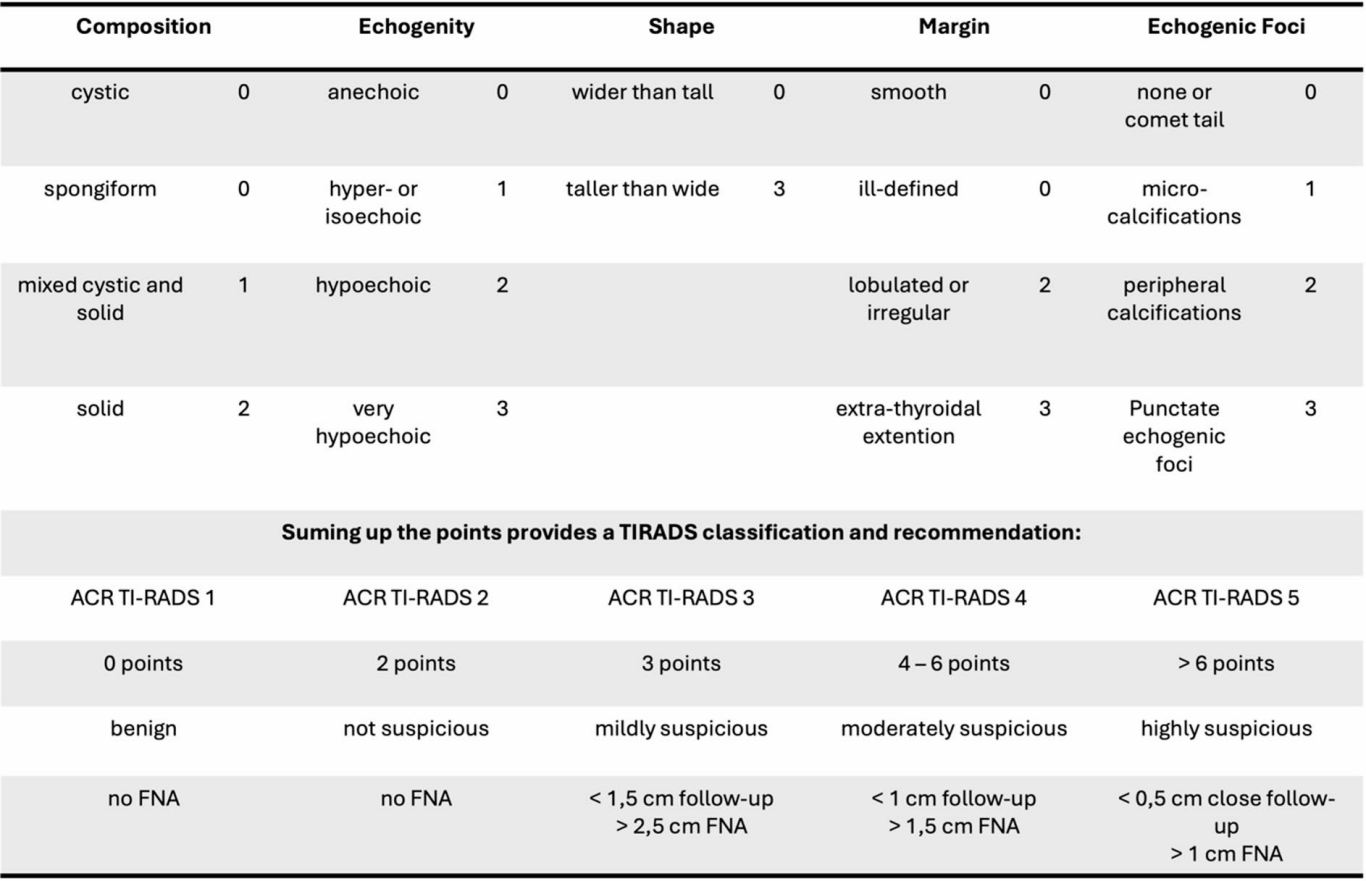
The AI-assisted software is based on the risk stratification system of the American college of radiology (ACR TI-RADS), modified from [5]

Patient treatment was solely based on the consultant’s final assessment. Surgical residents rotate through the specialist units of the department, so 7 different surgeons-in-training generated ultrasound images in the outpatient clinic; however, the bulk of the data was generated by 3 residents, the first author among them.

Via hospital case number, all ACR TI-RADS assessments of the assessor with the corresponding classification generated by the AI-assisted ultrasound were recorded together with the corresponding histopathological results in cases in which the patients underwent surgery. After each of several software updates delivered by the medical device company during the study period, the saved case files were re-run with the newly trained AI-assisted software. Anonymous Mainz ultrasound scan data files together with corresponding TI-RADS assessments of the assessor and histopathological results - as verification of the truly benign or malignant nature of the thyroid nodules - was used by the medical device manufacturer together with clinical data from other sources to improve the general software package. From March 2024 onwards, therefore, an improved software version was available.

In the second study phase, the software thus improved by deep learning was used in the outpatient clinic. The saved thyroid scans of the initial patients were re-run with the AI-system and re-assessed by the software. New patients were sonographed, so that a total of 133 patients with 228 nodules were included in this second study phase.

**Fig. 3:**
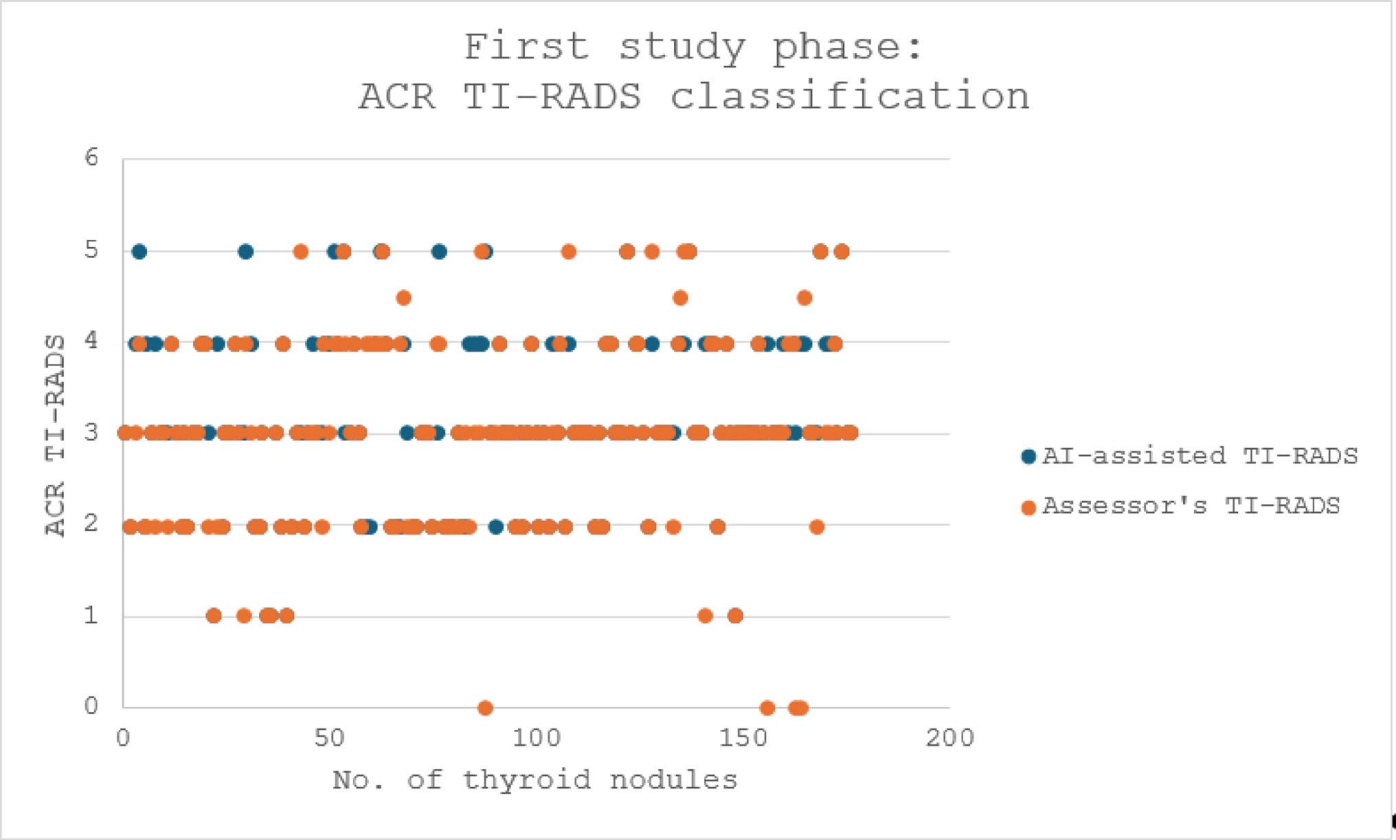
Before the AI training: comparison between the preoperative thyroid nodule risk stratification as assessed by the AI- software versus the assessor

**Fig. 4:**
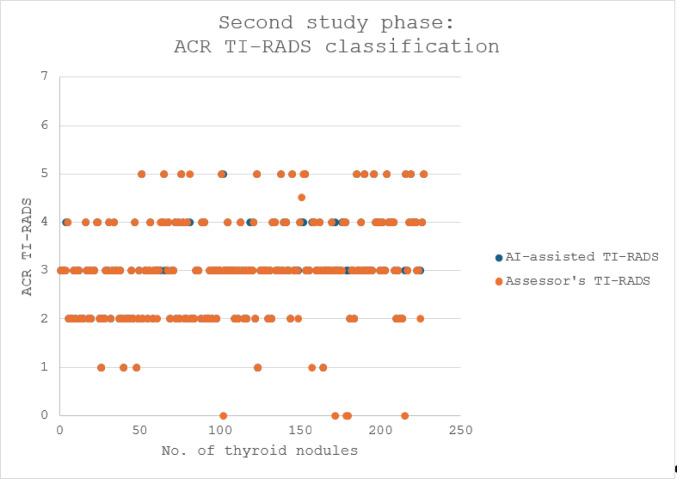
After the AI training: comparison between the preoperative thyroid nodule risk stratification as assessed by the AI-software versus the experienced assessor

An analysis of the accuracy of the AI-assisted ultrasound software was carried out (Figs. 3 and 4).

## Results

The judgement of the assessor was used as Gold Standard in our study, that is, his preoperative assessment of the ACR TI-RADS points was set as “true” and revised only in cases with contradictory histopathological results after performed (hemi)thyroidectomy. This reflects the real-world clinical situation where a single experienced senior clinician/surgeon evaluates the diagnostic neck sonography and decides on the indication (or lack thereof) for thyroid surgery.

Among the 57 patients who underwent thyroid surgery (Table 1), histopathological results were available as a means of verification of the preoperative risk assessments of the thyroid nodules (Table 3).

**Table 3:** Pathology results vs. assessor’s preoperative ACR TI-RADS classification

ACR TI-RADS	Histopathological finding	No. of nodules	Notes
1–3	Benign lesions	25	
3	PTC	3	2 microcarcinomas
4	PTC MTC Benign lesions	7 1 19	4 toxic nodules 4 Hashimoto thyroiditis 1 granulomatous nodules (likely Tbc) 1 FNAB false-positive (suspicious for PTC, BRAF V600E-negative)
5	PTC MTC PDTC/Anaplastic Benign	12 1 1 4	1 Hashimoto thyroiditis, 1 contralateral to BRAF V600E-positive PTC

## First study phase

The AI-assisted software identified and assessed 176 nodules in 110 patients transferred for evaluation of thyroid disease. These represent not only simple thyroid nodules, but also several complex cases, varying from cysts to node conglomerates.

128 (72.7%) nodules were ACR TI-RADS classified by the AI-software precisely as by the assessor (*p-value = 0*,*0001; Cohen’s Kappa κ = 0*,*6203*).

small differences (1 to 3 points difference) were encountered in 36 thyroid nodules (20.45%).

relevant differences (over 3 points) have been noted in only 12 nodules (6.8%).

Relevant differences have only been noted in less than 7% of cases. Besides difficulties in interpreting inhomogeneous inflamed thyroid tissue, another important issue were nodule conglomerates. Selecting and analyzing each nodule individually was suboptimal, not allowing for a distinct analysis of each nodule, with some important smaller characteristics (i.e. echogenic foci, microcalcifications) being overlooked by the software.

Sequential software updates and iterative re-analysis of stored imaging data improved performance notably (Table 4).

**Table 4:** The AI–assessor congruency improved notably over time, rising from 63–73% in early cohorts to 97% in the final group, reflecting the AI system’s progressive learning and software refinement

Patient group	No. of nodules	Congruent assessments	Small differences	Relevant differences
1–30	51	37 (72.5%)	10 (19.6%)	4 (7,8%)
31–56	46	29 (63%)	12 (26%)	5 (11%)
57–89	47	41 (87.2%)	4 (8.6%)	2 (4.2%)
90–110	32	23 (72%)	5 (15.6%)	4 (12.5%)
111–133	33	32 (97%)	0 (0%)	1 (3%)

## Second study phase

A major software package update was provided by the medical device manufacturer; the AI-assisted software had been improved by deep learning procedures performed with verified clinical data of different sources including the Mainz data.

The congruency between the assessor’s TI-RADS classifications and the assessments of the updated AI-assisted software notably improved in the new patients examined:

- The latest 23 patients included in the study had a total of 33 nodules, with a congruency of 32/33 (97%) while in only 1/33 (3%) a significant difference was noted.

In this second phase of the study, also all saved raw data from the thyroid scans were once again analyzed with the updated version of the AI-software. Thus, in the initial 110 patients, 194 nodules were analyzed, compared to the initial 176, as the software was better able to separate nodules within conglomerates and correctly assess them.

Combining the data of all examined patients, the updated software analyzed as follows (Fig. 4):

- patients 1–133 presented total of 227 nodules:
  - 210/227 (92.6%) were correctly assessed;
  - in 11 nodules (4.8%), small differences were noted between the AI and assessor’s classifications;
  - relevant differences were noted in 6 nodules (2.6%).

**Fig. 5:**
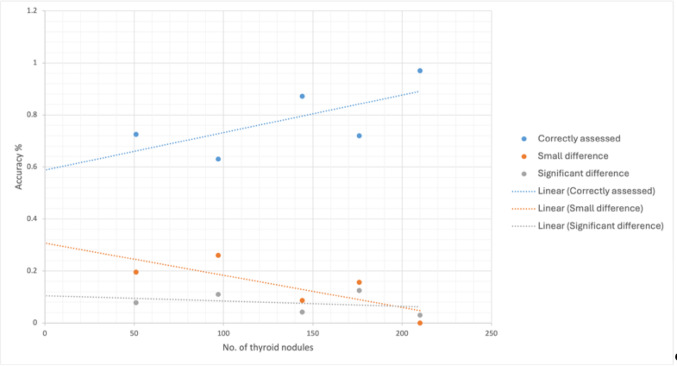
The accuracy of the AI-software increased steadily after data collection, several rounds of AI-training and software update, from an initial 72.5% (0.72) to 97% (0.97) in the latest nodules scanned

With the updated software, especially the better detection and analysis of each separate nodule resulted in an improved congruency in the TI-RADS classifications of the AI-assisted software with the judgement of the assessor (*p-value = 0*,*087; Cohen’s Kappa κ = 0*,*8955*). Furthermore, an improved analysis of nodules in inflamed thyroid tissue was noted. Not a single nodule was poorer assessed by the updated version compared to the initial software version. The fast “learning curve” of the PIUR-tUS Infinity software is depicted in Fig. 5.

Discrepancies were mainly related to Hashimoto thyroiditis and non-nodular findings, highlighting limitations of AI-assisted analysis in differentiating inflammatory or cystic structures from true nodules (Table 5).

**Table 5:** Description of cases with discrepancies between AI and expert TI-RADS classifications after updates

	No. of nodules	Assessor’s TI-RADS	AI-TI-RADS	Relevant differences
Minor discrepancies - no clinical impact	11	2 2 3 3 4 5 5 4 4 3 5	3 3 4 4 3 4 4 3 5 4 4	Unremarkable findings Thyroiditis Mismatch Toxic adenoma Mismatch Thyroiditis Thyroiditis
Major discrepancies - clinically relevant	6	0 0 0 0 1 0	3 3 3 4 4 5	Thyroiditis, no nodules Thyroiditis, no nodules Thyroiditis, no nodules Thyroiditis, no nodules Haemorrhaged cyst Intrathyroidal Thymus

## Discussion

### Training of AI-assisted software for thyroid nodule risk stratification

This study set out to investigate the effectiveness of training and deep learning application in the sonographic diagnosis and preoperative risk assessment of thyroid nodules, including the evaluation of diagnostic accuracy and context-dependence of the artificial intelligence (AI)-assisted ultrasound software used.

The raw thyroid ultrasound image data collected routinely in an outpatient clinic was accompanied by clinical information as available: age, sex, genetics of FNABs, present thyroid disease such as Hashimoto or Graves/Basedow etc., and especially by the histopathological results of those patients who underwent thyroid resections. While the histopathological diagnosis of malignancy (or not) of an examined nodule is irrefutable, the true nature of a thyroid nodule that was not subjected to a histopathological examination can of course only be judged in a subjective manner, namely, by an expert. In our study, the expert (“assessor”) was an experienced endocrine surgeon who has examined all his patients with neck complaints (thyroid, parathyroid, trachea, paraganglioma etc.) by sonography himself for decades, and has therefore seen plenty of unusual sonographic findings and remembers their post-operative outcomes or follow-ups. Naturally, an expert judgement that a specific sonographic feature of a thyroid nodule does not require surgery can only be verified by a long-term follow-up of this nodule. Examples are an intrathyroidal non-descended thymus in a child or a hemorrhaged cyst, which will both regress over time; or a benign thyroid nodule which will not grow or develop signs of malignancy for years. Quite often, nevertheless, a patient becomes weary of yearly follow-ups and asks for a definite nodule assessment by surgery and histopathology, so that the surgeon is finally reassured of his initial (benign) judgement. With confidence in the suitability of the expert opinion used in our study, we set his assessments of the ACR TI-RADS points of a specific thyroid nodule as “true”.

After several rounds of minor software updates and “training the AI”, the diagnostic accuracy of the software increased, with a fast and steady learning curve (Fig. 5). After implementation of a new software version after deep learning application by the medical device company (with more data used than just the Mainz data), a significant improvement in correct TI-RADS classification was demonstrated, from an initial 72.5% to 97% diagnostic accuracy in the latest nodules scanned. Of course, for the Mainz clinic there is a bias in this improvement, since all corrected Mainz assessments will now be stating the “correct” ACR TI-RADS as judged by the assessor or by histopathology. However, the software learned, and compared nodules of new patients examined to those already corrected for risk assessment, and came to improved results. Notably, the assessment of nodules within conglomerates improved.

### Difficulties encountered by AI-assisted software for thyroid nodule risk stratification

At the beginning of our study, AI misinterpreted nodules with microcalcifications or echogenic foci, leading to 1–3 points differences to the accessor’s risk stratification. After software update, the recognition of these nodule features improved. Other difficulties noticed were correct identification and risk stratification of cysts or very hypoechogenic nodules.

Initially, the used AI-assisted software system displayed less accurate assessments of nodules in patients with autoimmune disease of the thyroid, where separating the inhomogeneous thyroid tissue from specific nodules proved somewhat challenging for the software. However, this is also challenging for clinicians; user experience in thyroid ultrasound makes a difference; and the cases in which our expert was contradicted by histopathology in his TI-RADS assessments of nodules were overproportionally nodules in thyroiditis patients. However, AI-software has proven effective in diagnosing nodules in Hashimoto thyroiditis before [8], and the diagnostic accuracy of the software we used improved after software update, indicating at the ability of the software to better perform by collecting and analyzing more data, and by allowing deep learning to occur.

## Quality and diversity of datasets used to train AI

Successful AI training in medical devices, especially with deep learning application, does not only need large raw image data sets ideally collected by different clinics with patient heterogeneity (data diversity), but is dependent on clean, verified data (data quality). In case of thyroid nodule risk stratification, optimal verification of course is achieved by histopathology, either of the nodule that was judged high-risk (ACR TI-RADS 4 or 5) and resected to rule out malignancy, or of the benign nodule that was a side-finding in a surgical specimen. Second-best is years of thyroid sonographic follow-up information to verify the judgement of TI-RADS 1 and 2 nodules. The cases in which this data is available are limited, however. A sufficient quantity of data on unusual pathology (example: lymphoma in thyroid tissue) or rare abnormal findings (example: intrathyroidal thymus) can be collected only over time and by multiple institutions.

Several authorities warn that overfitting to training data is a potential weakness that could severely impair the performance of deep learning systems in real-world applications. It means that the extremes in the Gaussian distribution or unusual clinical findings that do not fit in the programmed categories are removed by the training module. Differently structured or unrepresentative datasets can lead to reduced performance, highlighting the need for careful data selection [24–26]. Some researchers demonstrate that heterogeneous datasets produce the best results by training the algorithms against a wide range of samples, while others point to the importance of data preprocessing to minimize noise and bias [25]. In a study analyzing the AI-human interaction, with several less experienced physicians/examiners, there was a significant difference in output accuracy (between 40% and 100%) depending on the examiner [27].

## User friendliness of the AI-assisted system for thyroid sonography

The PIUR tUS Infinity software system provides an intuitive interface, making it user friendly even for less experienced thyroid sonographers. After a short learning curve for the users (in our outpatient clinic: rotating residents or fellows, whether or not interested in pursuing a career in endocrine surgery), they managed to implement the AI-assisted ultrasound diagnostic tool in our routine, with only little increasing examination time per patient. The slight plus in time was not due to the conscientious filling out of the ACR TI-RADS form which would be the same in case of use of a printed form version. Rather, the manual correction of the automated circular marking of the nodule boundaries in 3 dimensional planes (for the construction of the 3D gland) is more time-consuming than a usual ultrasound scan. An improvement in skills and therefore in the quality of the 3D thyroid scans was rapidly noted of the beginners, with quite some artefacts and somewhat poorer results in the very first patients, and rapid improvement already after a few further patients examined.

### Key features of the AI-assisted software system used in our study

**Fig. 6:**
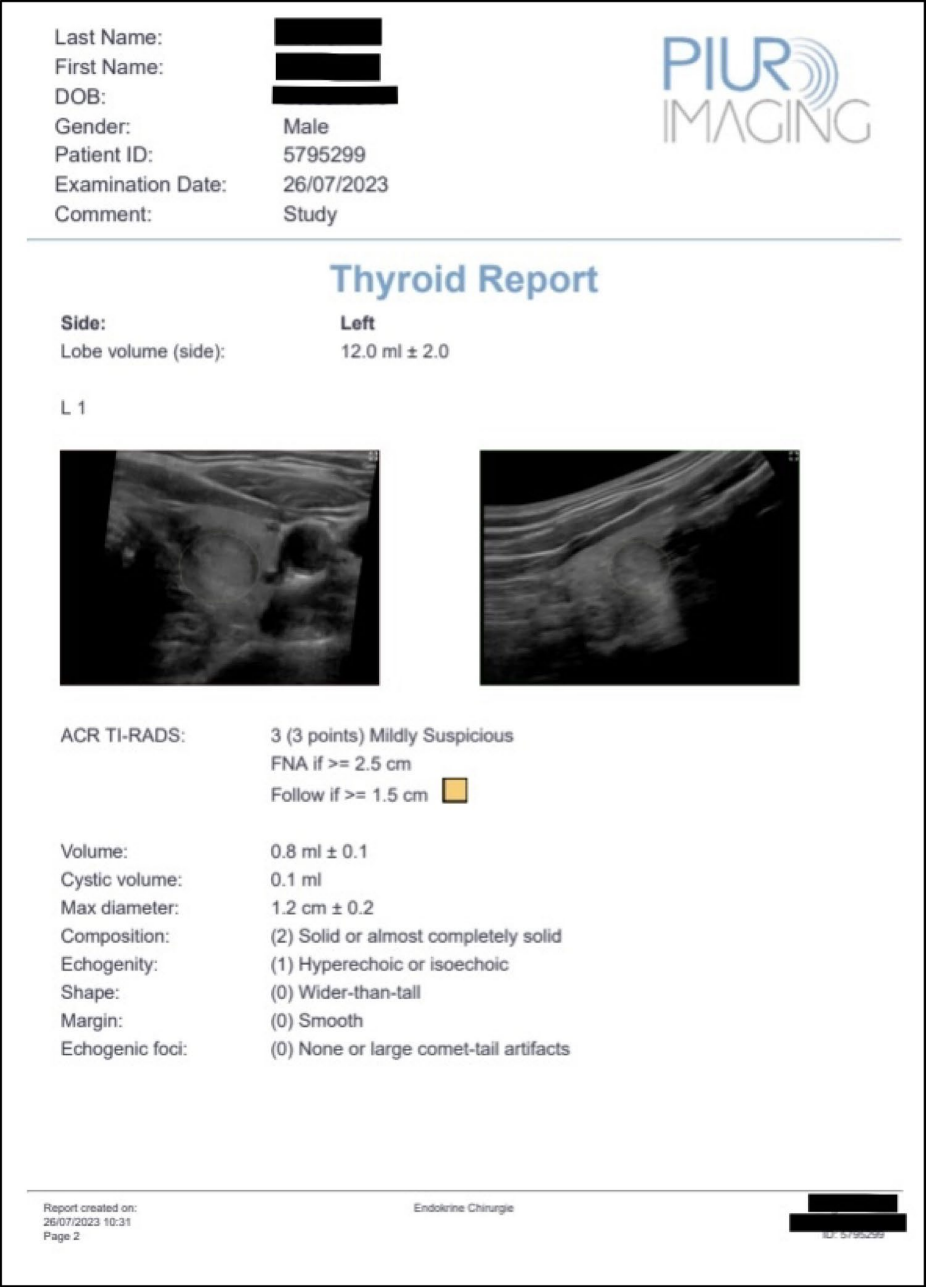
Example of an AI-assisted thyroid ultrasound examination report

- real-time imaging: the software allows users to view images in real time, offering immediate feedback, enabling a fast assessment and adjustments during the scanning process;
- 3D visualization: a 3D model of the thyroid lobe and of the nodules harbored within is provided. This feature allows for better spacial recognition and for differentiation of overlaying nodules, thereby ensuring a good understanding of the anatomy of the examined gland (later the surgical field);
- automated nodule recognition: this feature provides a solid support for users, ensuring that anomalies within the thyroid nodules are noticed, reducing the likelihood of human error;
- ACR TI-RADS classification: the application of any risk stratification system for thyroid nodules can seem overwhelming for beginners who still struggle with finetuning of the thyroid sonography itself. The ACR TI-RADS nodule risk stratification form integrated in the software and the automated adding up of the risk score, as well a clear representation of the nodule characteristics which led to this score, is a significant support for the user;
- user control and software flexibility: while the software provides a solid automated process, it also allows for adjustments by the user, when deemed necessary;
- result reporting meeting medico-legal requirements: the patient ID and the results of an examination - that is, thyroid and nodule descriptions, dimensions and volumes, ACR TI-RADS classification etc. – can be printed, saved on the machine, or transferred in DICOM format (digital imaging and communications in medicine) into the hospital documentation system (Fig. 6). The saved data file can be opened again at a later time-point and compared to the actual scan of the same patient, so that even slight nodule growth or progressions in suspect features can be noticed. Saved data files can be re-evaluated after software updates;

- automated recommendations for further diagnostics: dependent on the nodule features or TI-RADS score, the software provides suggestions for FNAB, clinical follow-up etc., thereby giving food for thought to a less experienced sonographer;

Especially the software features automated nodule recognition, correct identification of TI-RADS-relevant findings such as calcifications, and the automated clinical recommendations depend on AI training and deep learning procedures.

### Role of AI-assisted software for thyroid nodule risk stratification in teaching

The benefits of using AI-assisted imaging in clinical practice surpass patient care: studies already report on benefits in incorporating AI-assisted software in training of students and young physicians [22]. We observed that the use of the PIUR tUS Infinity software forced the rotating residents (or fellows) - who do not necessarily have a pointed interest in endocrine surgery - to learn and memorize a risk stratification system for thyroid nodules. In our outpatient clinic, we followed the procedure that the residents sonographed with the AI-assisted software the patients first - in the presence of a wall hanging depicting the ACR TI-RADS classification with photographed nodule examples—and printed out their own assessment. Then they observed the sonography done by the assessor (without software assistance) and listened to his decision on the ACR TI-RADS risk score; finally, divergent assessments were discussed which provided an excellent learning opportunity.

Thyroid surgery is one of the most often performed surgical procedures, so the surgeons-in-training will certainly set their knowledge gained in thyroid sonography to good use in their future. A side-effect is that they gain the insight that going through a list of diagnostic features in a structured fashion (in this case: a risk assessment form [28]) increases the diagnostic certainty in numerous clinical decision-making settings. Especially the recommendation of an elective surgery to a patient should be based on solid argumentation.

Danger of overreliance on automated diagnostic systems by unexperienced examiners.

Numerous studies highlight the possibilities offered by AI technologies for diagnosing thyroid diseases, particularly through the analysis of existing sonography data. It is certain that AI performs similarly to human experts in detecting anomalies in thyroid disease [3–7, 16–26]. Nevertheless, the reliability and generalizability of these models in all possible clinical situations remains a critical issue; the user always must critically scrutinize the merit of the AI-assisted software’s assessments and recommendations. Naturally, this is more difficult for physicians-in-training than for experienced clinicians. In a study analyzing the clinical value of using AI-software in assisting junior radiologists, the AI-software improved the overall performance, yet misled physicians-in-training in a few cases of unusual/abnormal findings [29]. In clinics without experts to support inexperienced users, it is especially crucial to use optimally trained AI systems.

In summary, AI-assisted ultrasound systems are already a solid tool in the diagnosis of true thyroid nodules in non-inflamed tissue. AI-software cannot yet replace an experienced clinician or endocrine surgeon in complex or unusual cases, but the fast learning curve demonstrated by the system used in our study after several rounds of deep learning is indeed encouraging.

## Data Availability

Due to anonymous retrospective data processing of routine diagnostic data
